# Trajectories of functional and structural myocardial parameters in post-COVID-19 syndrome—insights from mid-term follow-up by cardiovascular magnetic resonance

**DOI:** 10.3389/fcvm.2024.1357349

**Published:** 2024-04-02

**Authors:** Jan Gröschel, Leonhard Grassow, Phillip van Dijck, Yashraj Bhoyroo, Edyta Blaszczyk, Jeanette Schulz-Menger

**Affiliations:** ^1^ Charité—Universitätsmedizin Berlin, Corporate Member of Freie Universität Berlin and Humboldt-Universität zu Berlin, ECRC Experimental and Clinical Research Center, Berlin, Germany; ^2^ Working Group on Cardiovascular Magnetic Resonance, Experimental and Clinical Research Center, a Joint Cooperation Between Charité Medical Faculty and the Max-Delbrück Center for Molecular Medicine, Berlin, Germany; ^3^ DZHK (German Centre for Cardiovascular Research), Partner Site Berlin, Berlin, Germany; ^4^ Deutsches Herzzentrum der Charité—Medical Heart Center of Charité and German Heart Institute Berlin, Klinik für Kardiologie, Angiologie und Intensivmedizin, Berlin, Germany

**Keywords:** cardiovascular magnetic resonance, COVID-19, post-COVID-19 syndrome, mapping, late gadolinium enhancement, stroke volume

## Abstract

**Introduction:**

Myocardial tissue alterations in patients with post-Coronavirus disease 2019 syndrome (PCS) are often subtle and mild. Reports vary in the prevalence of non-ischemic and ischemic injuries as well as the extent of ongoing myocardial inflammation. The exact relevance of these myocardial alterations is not fully understood. This study aimed at describing the trajectories of myocardial alterations in PCS patients by mid-term follow-up with cardiovascular magnetic resonance (CMR).

**Methods:**

This study entails a retrospective analysis of symptomatic PCS patients referred for follow-up CMR between August 2020 and May 2023 due to mildly affected or reduced left or right ventricular function (LV and RV, respectively) and structural myocardial alterations, e.g., focal and diffuse fibrosis, on baseline scans. Follow-up CMR protocol consisted of cine images and full coverage native T1 and T2 mapping. Baseline and follow-up scans were compared using *t*-tests or Wilcoxon tests. *Post-hoc* analysis was carried out in a subgroup based on the change of LV stroke volume (SV) between scans.

**Results:**

In total, 43 patients [median age (interquartile range) 46 (37–56) years, 33 women] received follow-ups 347 (167–651) days after initial diagnosis. A decrease in symptoms was recorded on follow-ups (*p* < 0.03) with 23 patients being asymptomatic at follow-ups [symptomatic at baseline 43/43 (100%) vs. symptomatic at follow-up 21/43 (49%), *p* < 0.001]. Functional improvement was noted for LV-SV [83.3 (72.7–95.0) vs. 84.0 (77.0–100.3) ml; *p* = 0.045], global radial [25.3% (23.4%–27.9%) vs. 27.4% (24.4%–33.1%); *p* < 0.001], and circumferential strains [−16.5% (−17.5% to −15.6%) vs. −17.2% (−19.5% to −16.1%); *p* < 0.001]. In total, 17 patients had an LV-SV change >10% on follow-up scans (5 with a decrease and 12 with an increase), with LV-SV, RV-SV, and global longitudinal strain being discriminatory variables on baseline scans (*p* = 0.01, 0.02, and 0.04, respectively). T1- or T2-analysis revealed no changes, remaining within normal limits.

**Conclusion:**

Symptomatic load as well as blood pressures decreased on follow-up. CMR did not detect significant changes in tissue parameters; however, volumetric, specifically LV-SV, and deformation indexes improved during mid-term follow-up.

## 1 Introduction

After the Coronavirus disease 2019 (COVID-19) pandemic impacted healthcare systems worldwide, the next challenge for physicians emerged in the form of the Post-COVID-19 syndrome (PCS) (1, 2). This term was introduced to categorize and characterize patients with prolonged complaints, defined by symptoms lasting for more than 12 weeks after an acute COVID-19 infection (1). The symptoms include fatigue, dyspnea, exercise intolerance, palpitations, and chest pain among other neurologic manifestations such as concentration difficulties and memory impairments (3). Outpatient cardiologists are often tasked to rule out an underlying cardiac pathology as the sequalae of an acute COVID-19 infection might include myocarditis, arrhythmias, myocardial infarctions, and heart failure (4). Basic work-up includes an electrocardiogram, laboratory assessments in the form of troponin and N-terminal pro b-type natriuretic peptide (NT-pro-BNP) levels as well as transthoracic echocardiography (2, 5). When abnormalities on this basic work-up testing are found, the next step is often a referral for cardiovascular magnetic resonance (CMR) as a recent study reported an increased frequency of myocardial structural impairments in this patient subgroup (6). Even in the absence of abnormalities, on basic work-up patients are often referred for further CMR diagnostics owing to ongoing symptoms (7). Current expert consensus recommends CMR to be carried out after a COVID-19 infection with ongoing symptoms as this modality can accurately assess biventricular function and further provide tissue quantification to detect focal changes, such as scars or fibrosis on late gadolinium enhancement (LGE), diffuse fibrosis on T1 mapping, and detection of active myocardial inflammation in the form of T2-weighted imaging or quantitatively by T2 mapping (7–9). Rates of abnormal findings on CMR vary depending on the severity of the initial infection as well as cardiovascular comorbidities and risks (10–12). In outpatient groups, myocardial alterations are either absent or subtle (13, 14). Reports vary in the prevalence of non-ischemic and ischemic injuries as well as the extent of ongoing myocardial inflammation (15). It is important to note that left ventricular (LV) function is often preserved and only indexes of myocardial deformation are impaired (14). There is a lack of evidence regarding patients with symptoms and mild myocardial involvement on baseline CMR. In addition, the exact relevance of these myocardial alterations on baseline is not fully understood and therefore remains to pose a diagnostic and therapeutic challenge. Recently an emphasis has been put on biventricular functional and volumetric parameters, which might potentially be impaired in patients with PCS (16). Subtle and mild alterations may often be missed and detailed follow-ups (FU) are needed. This study aimed to describe the trajectories of myocardial alterations in patients with PCS and slight alterations on baseline CMR by mid-term FU, focusing on functional, volumetric, and deformation indexes.

## 2 Material and methods

### 2.1 Study patients

For this exploratory study all patients referred for an outpatient FU CMR exam between August 2020 and May 2023 with ongoing symptoms after COVID-19 infection and mild alterations on baseline CMR scans were included and retrospectively analyzed. Alterations on baseline scans included reduced right ventricular (RV) (RV ejection fraction <42%) and/or LV function (LV ejection fraction <55%) and/or native T1 times >1.037 ms and/or T2 times >54 ms and/or focal fibrosis on LGE imaging. All cutoffs were defined based on established in-house post-processing and analysis with limits derived from healthy cohorts (17–19). The time of the acute event was defined by the first positive polymerase chain reaction test. Patients were excluded from final analysis if severe systemic illnesses, severe acute COVID-19 infection (including hospitalization for the acute infection), or previous chemotherapy were known. Finally, if arrhythmias during the scan impaired image acquisition or the examination was incomplete, patients were excluded. To compile basic characteristics, including medical history, drugs taken as well as previous imaging and laboratory assessment, documents from the outpatient providers as well as the local hospital records were considered.

#### 2.1.1 Ethical statement

This study complies with the Declaration of Helsinki and was approved by the institutional ethics committee. The requirement for written informed consent was waived due to the retrospective study design (EA1/042/22). The baseline results were presented in a previous publication (14).

### 2.2 CMR protocol

All CMR exams were acquired on a 1.5 T Scanner (AvantoFit, Siemens, Erlangen, Germany) with ECG-gating and a 32-channel phased-array surface coil. For biventricular function assessment, balanced steady-state free precession cine images were acquired in four long-axis views including a four-, two-, three-chamber view as well as an RV view and one short-axis (SAX) stack, covering the entire ventricle without a gap. Parametric T2 and T1 mapping were acquired in multiple SAX slices covering the entire ventricle. T2-mapping acquisition was based on a motion-corrected balanced steady-state free precession sequence. Native T1 mapping was based on a motion-corrected modified Look-Locker inversion recovery technique using a 5-3-3 scheme. Synthetic extracellular volume was calculated from T1 mapping pre- and post-contrast media application based on a prototype sequence in basal and mid-ventricular slices. LGE imaging was acquired by a phase-sensitive inversion recovery sequence, 10–15 min after the application of 0.2 mmol/kg of contrast media (Gadoteridol, Prohance, Bracco Imaging, Konstanz, Germany). LGE images were acquired in the same axis as the cine images. FU scans were carried out using the same protocol with the exception of a subgroup of patients not receiving contrast media (*N* = 27). The details about the sequence parameters are given in the Supplemental Material E1.

### 2.3 Data analysis

Two readers [one with 7 years of experience in CMR (YB) and one resident in-training with 3 years of experience (JG)] performed image analysis using CVI42 (version 5.13.0, Circle Cardiovascular Imaging, Calgary, Canada). LV and RV function assessments were executed on cine SAX images following current recommendations regarding the papillary muscle mass attributed to the total LV mass (20). Right atrial function was assessed on the cine four chamber view. Left atrial function was assessed biplanar using the two- and four- chamber views. Strain analysis was carried out with retrospective feature tracking on cine images as reported recently (21). Global values for longitudinal, radial, and circumferential strain are reported. Quantitative mapping analysis was carried out with endo- and epicardial border delineations in each slice to obtain global values. Slice locations were allocated in the respective segment and level by delineating the extent of the LV. Slices with visible LV-outflow tracts were excluded. Similarly, apical slices with no blood pool or thin myocardial walls were excluded. A qualitative survey ensured the exclusion of segments with artifacts as well as focal fibrosis detected by LGE. Focal scars were assessed visually by LGE analysis by both readers independently concerning presence and location of scars. In case of uncertainties, a consensus read was performed.

### 2.4 Statistical analysis

Continuous variables are expressed as median and interquartile range (IQR) or mean and standard deviation (SD) as appropriate. Categorical variables are given as absolute frequencies and percentages. Normal distribution was assessed by the Shapiro–Wilk test. Baseline and FU scans were compared using either the *t*-test or the Wilcoxon rang test for continuous variables and the *χ*^2^ or Fisher's exact test for categorical variables. A *post-hoc* group analysis based on the percent change of LV stroke volume (LV-SV) (increase >10% vs. decrease >10% vs. change <10%) was carried out with either ANOVA or the Kruskal–Wallis test as global tests and followed-up in the cases with significant findings with pairwise testing. As the analysis was regarded as exploratory in nature, no corrections for multiple tests were carried out. A *p*-value <0.05 was defined as being statistically significant whereas a *p*-value <0.10 was considered as a trend. Statistical calculations were performed using the software SPSS Statistics (Version 27.0.0, IBM, Armonk, NY, USA).

## 3 Results

### 3.1 Patient characteristics

In total, 43 patients [median age (IQR) 46 (37–56) years, 33 women, height 167 cm (163–180 cm), weight 70.0 kg (63.0–82.5 kg), body mass index 25.0 (22.1–27.0)] received FUs. Baseline CMRs were carried out at an average of 155 (70–239) days after acute infection with FUs having occurred at an average of 347 (167–651) days after initial diagnosis and 155 (56–440) days after baseline CMR. Alterations on baseline scans were as follows: 5 patients displayed reduced LV function, 1 displayed reduced RV function, 14 showed T1 times >cutoff, 4 showed T2 times >cutoff, and 20 had focal scars. On FU scans, patients displayed lower systolic [baseline 123 mmHg (118–130) vs. FU 110 mmHg (100–120); *p* < 0.001] and diastolic blood pressure [baseline 74 mmHg (67–83) vs. FU 68 mmHg (62–76); *p* = 0.01] with no significant difference in heart rate between scans [baseline 74 beats per minute (72–81) vs. FU 68 beats per minute (67–82); *p* = 0.57]. Overall comorbidity burden was low with arterial hypertension [9/43 (21%)] and hyperlipidemia [4/43 (9%)] being the most frequent conditions. The remaining comorbidities included diabetes mellitus [2/43 (5%)] and non-COVID-19-related lung diseases [3/43 (7%)]. No patient had a history of coronary artery disease, myocardial infarction, heart failure with reduced ejection fraction, valvular heart disease, or arrhythmias. At baseline, fatigue [27/43 (63%)] and dyspnea [31/43 (72%)] were reported as the leading symptoms. The overall symptom load declined between baseline and FU scans [total symptomatic patients at baseline 43/43 (100%) vs. FU 21/43 (49%), *p* < 0.001] as well as the symptoms themselves (*p* for all symptoms < 0.03). This, however, was not accompanied by a change in drug regimen. Detailed drug treatment and symptoms at baseline and FU are presented in Table 1.

**Table 1:** Drug treatment and symptoms in the PCS cohort.

Parameter	Baseline (*N* = 43)	Follow-up (*N* = 43	*p*-value
Medications			
Angiotensin converting enzyme inhibitors/angiotensinogen II receptor blockers	11/43 (26%)	11/43 (26%)	>0.99^a^
Beta blockers	11/43 (26%)	11/43 (26%)	>0.99^a^
Mineralocorticoid antagonists	1/43 (2%)	0/43 (0%)	>0.99^b^
Thiazide diuretics	1/43 (2%)	1/43 (2%)	>0.99^b^
Calcium channel blockers	4/43 (9%)	4/43 (9%)	>0.99^b^
Statins	2/43 (5%)	2/43 (5%)	>0.99^b^
Insulin	1/43 (2%)	1/43 (2%)	>0.99^b^
Metformin	1/43 (2%)	1/43 (2%)	>0.99^b^
Symptoms			
Total symptomatic patients	43/43 (100%)	21/43 (49%	**<0**.**001**^a^
Fatigue	28/43 (65%)	8/43 (19%)	**<0**.**001**^a^
Dyspnea	32/43 (74%)	8/43 (19%)	**<0**.**001**^a^
Chest pain	18/43 (42%)	7/43 (16%)	**0**.**01**^a^
Palpitations	20/43 (47%)	10/43 (23%)	**0**.**02**^a^

Values presented as absolute and percent. A *p*-value <0.05 was considered significant; *p*-values in bold represent significant findings.

a *χ*^2^ test.

^b^ Fisher's exact test.

### 3.2 Additional tests

Given the retrospective nature of the study, only a minority of patients reported laboratory results at baseline (*N* = 23) with even less at FU (*N* = 5). At baseline, one patient had a NT-pro-BNP above the laboratory cutoff (>125 ng/L) with the rest not revealing any abnormalities [NT-pro-BNP: 70 ng/L (39–108); Troponin T high sensitivity 3 ng/L (3–7)]. Baseline ECGs revealed a first-degree atrioventricular (AV) block in one patient. Additional ambulatory long-term ECG recordings (between 24 and 48 h) at baseline were available in 18 patients. One patient had a short non-sustained ventricular tachycardia. Other recordings did not show any abnormalities.

### 3.3 CMR results

**Figure 1:**
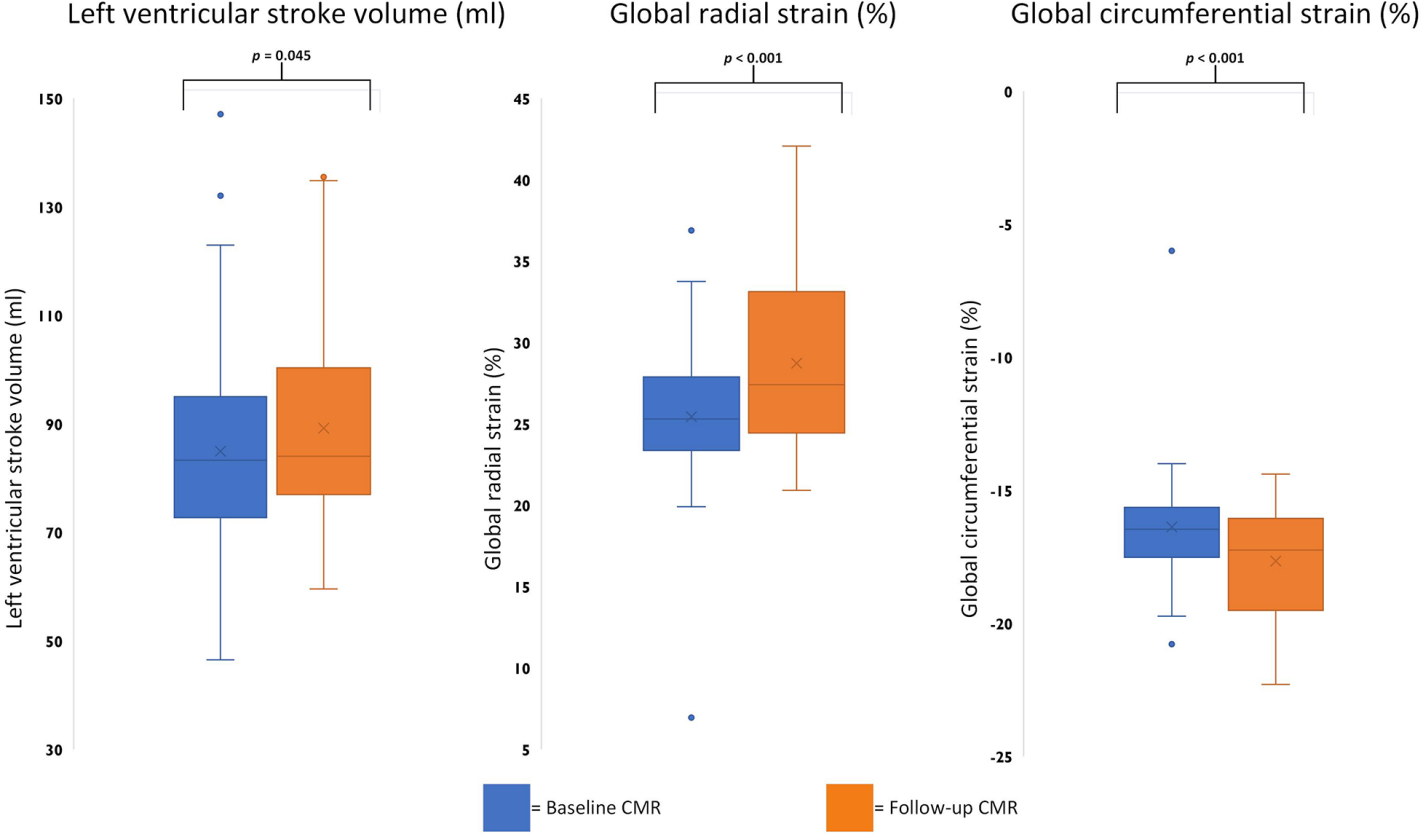
Boxplots comparing significant baseline and follow-up CMR parameters. Boxplots representing the median (solid inside the box), interquartile range (box), and 1.5 × interquartile range (whiskers) for left ventricular stroke volume, global radial strain, and circumferential strain (left to right) for baseline (blue) and follow-up (orange) scans. Every value below or above 1.5 × interquartile range is marked as an outlier.

Statistically significant improvement of function parameters was noted for LV-SV [baseline 83.3 (72.7–95.0) ml vs. FU 84.0 (77.0–100.3) ml; *p* = 0.045] with positive trends for LV ejection fraction and end-diastolic volume. Of the five patients with reduced LV ejection fraction on baseline exams, two showed a normalized LV ejection fraction on FU, whereas three remained below the cutoff of 55%, despite an overall improvement in LV ejection fraction. Overall RV function and volume remained stable without trends across the examined time frame. However, one patient with a reduced RV ejection fraction on baseline continued to have an impairment on FU. Atrial function did not differ between scans, and LV strain analysis revealed improvements for global radial [25.3% (23.4%–27.9%) vs. 27.4% (24.4%–33.1%); *p* < 0.001] and circumferential strains [−16.5% (−17.5% to −15.6%) vs. −17.2% (−19.5% to −16.1%); *p* < 0.001] (Table 2 and Figure 1).

**Table 2:** CMR results—total cohort.

Parameter	Baseline CMR (*N* = 43)	Follow-up CMR (*N* = 43)	*p*-value
LA (cm^2^)	18.8 (16.6 to 21.0)	19.4 (17.6 to 23.2)	0.13^a^
LA-EDV/BSA (ml/m^2^)	31.0 (27.7 to 36.9)	32.7 (27.2 to 39.1)	0.25^a^
LA-EF (%)	65.2 (61.9 to 69.3)	64.7 (60.1 to 67.9)	0.98^a^
RA (cm^2^)	19.6 (17.6 to 22.0)	20.4 (17.5 to 22.5)	0.37^a^
RA-EF (%)	46.3 (39.5 to 59.9)	47.7 (37.8 to 55.0)	0.54^b^
LV-EDV (ml)	134.7 (121.7 to 147.1)	136.1 (121.0 to 158.3)	0.12^a^
LV-ESV (ml)	50.3 (42.7 to 58.6)	51.6 (42.3 to 60.8)	0.25^a^
LV-SV (ml)	83.3 (72.7 to 95.0)	84.0 (77.0 to 100.3)	**0**.**045**^a^
LV-EF (%)	63.5 (58.4 to 65.4)	64.2 (60.0 to 68.1)	0.07^a^
LV-M (g)	75.1 (63.2 to 89.2)	74.9 (66.3 to 96.8)	0.07^a^
RV-EF (%)	53.9 (50.5 to 58.0)	55.0 (51.5 to 57.0)	0.46^b^
RV-EDV (ml)	147.7 (127.3 to 163.1)	144.7 (131.6 to 172.9)	0.41^b^
RV-SV (ml)	78.5 (72.7 to 88.3)	77.3 (69.3 to 97.2)	0.32^b^
Global native T1 (ms)	1,015.7 (996.9 to 1,040.6)	1,018.2 (991.9 to 1,032.9)	0.24^a^
Cases T1 > cutoff (1,037 ms)	14/43 (33%)	8/43 (19%)	0.14^c^
Global T2 (ms)	49.8 (48.8 to 51.8)	49.9 (48.8 to 51.2)	0.83^a^
Cases T2 > cutoff (54 ms)	4/43 (9%)	0/43 (0%)	0.12^c^
Global ECV (%)	23.8 (22.7 to 26.5)	23.1 (22.4 to 25.5)	0.75^b^
Global longitudinal strain (%)	−18.6 (−19.7 to −17.1)	−18.1 (−19.3 to −16.7)	0.19^a^
Global radial strain (%)	25.3 (23.4 to 27.9)	27.4 (24.4 to 33.1)	**<0**.**001**^a^
Global circumferential strain (%)	−16.5 (−17.5 to −15.6)	−17.2 (−19.5 to −16.1)	**<0**.**001**^a^

LA, left atrium; LA-EDV, left atrial end-diastolic volume; BSA, body surface area; LA-EF, left atrial ejection fraction; RA, right atrium; RA-EF, right atrial ejection fraction; LV-EDV, left ventricular end-diastolic volume; LV-ESV, left ventricular end-systolic volume; LV-SV, left ventricular stroke volume; LV-EF, left ventricular ejection fraction; LV-M, left ventricular mass; RV-EF, right ventricular ejection fraction; RV-EDV, right ventricular end-diastolic volume; RV-SV, right ventricular stroke volume.

Values presented as median and IQR. A *p*-value <0.05 was considered significant; *p*-values in bold represent significant findings

^a^ Wilcoxon test.

^b^ Paired Student’s *t*-test.

^c^ *χ*^2^ or Fisher's exact test.

Tissue characterization by CMR showed no changes between baseline and FU for T1 and T2 mapping. Although none of the four patients with initially elevated T2 times showed these elevations on FU, no statistic trends were detectable. On baseline scans, 20 patients had positive LGE (1 in an ischemic pattern and 19 in a non-ischemic pattern). In a subgroup with contrast media application (16 patients) on FU, LGE as well as extracellular volume assessment were carried out. Extracellular volume showed no change over the time period studied, nevertheless, focal fibrosis on LGE showed a trend toward regression [baseline 8/16 (50%) vs. FU 3/16 (19%); *p* = 0.06]. The patient with the non-sustained ventricular tachycardia had no LGE.

### 3.4 Subgroups with changes in left ventricular stroke volume

**Figure 2:**
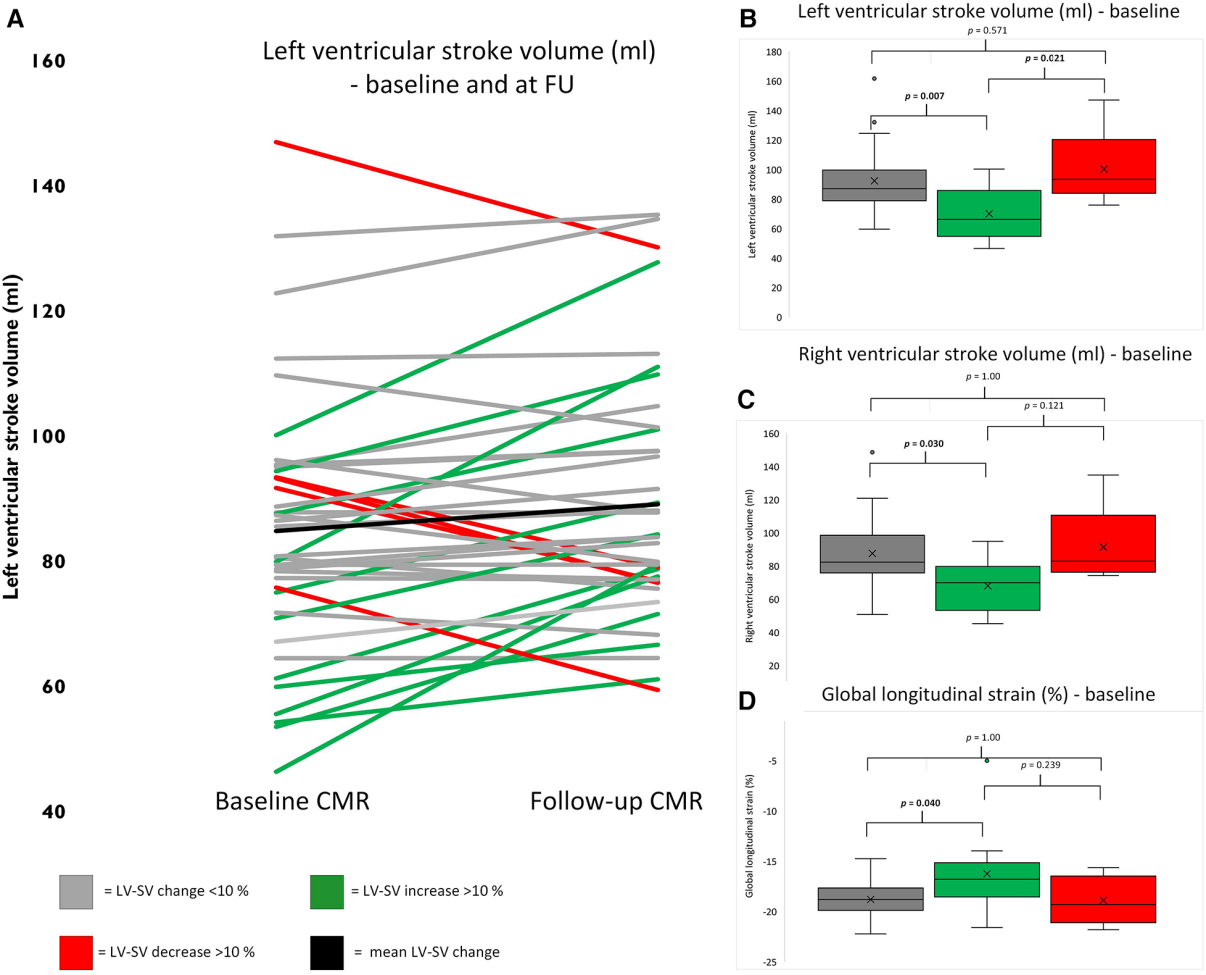
Trajectories for individual patients based on left ventricular stroke volume changes. (**A**) represents trajectories for LV-SV for each individual patient between baseline and follow-up scan. (**B**) depicts baseline values for left ventricular stroke volume, (**C**) for right ventricular strain, and (**D**) for global longitudinal strain. The boxplots represent the median (solid inside the box), interquartile range (box), and 1.5 × interquartile range (whiskers). Every value below or above 1.5 × interquartile range is marked as an outlier. Black, mean change; gray, change <10%; red, decrease >10%; and green, increase >10%.

In total, 17 patients had a change >10% of LV-SV on FU scans (5 with a decrease, 12 with an increase). Comparing the baseline variables between the subgroups, LV-SV, RV-SV, and global longitudinal strain were significantly different (Figure 2). Interestingly, patients with an improvement in LV-SV >10% on FU CMR had the lowest global longitudinal strain on baseline scans [patients with a change <10% in LV-SV −18.8% (−19.9% to −17.7%) vs. decrease >10% in LV-SV −19.3% (−21.1% to −16.5%) vs. increase >10% in LV-SV −16.8% (−18.6% to −15.2%); *p* = 0.04] (Table 3).

**Table 3:** Baseline characteristics of subgroups with left ventricular stroke volume changes.

Parameter	Change <10% of LV-SV (*N* = 26)	Increase >10% of LV-SV (*N* = 12)	Decrease >10% of LV-SV (*N* = 5)	*p*-value
Total symptomatic patients on baseline	26/26 (100%)	12/12 (100%)	5/5 (100%)	>0.99^a^
Fatigue on baseline	17/26 (65%)	9/12 (75%)	2/5 (40%)	0.39^a^
Dyspnea on baseline	18/26 (69%)	9/12 (75%)	5/5 (100%)	0.78^a^
Chest pain on baseline	10/26 (39%)	8/12 (67%)	0/5 (0%)	0.14^a^
Palpitations on baseline	12/26 (46%)	5/12 (42%)	3/5 (60%)	0.79^a^
Total symptomatic patients on FU	12/26 (46%)	6/12 (50%)	3/5 (60%)	0.65^a^
Fatigue on FU	3/26 (12%)	5/12 (42%)	0/5 (0%)	0.11^a^
Dyspnea on FU	4/26 (15%)	2/12 (17%)	2/5 (40%)	0.80^a^
Chest pain on FU	5/26 (195)	2/12 (17%)	0/5 (0%)	0.90^a^
Palpitations on FU	7/26 (27%)	1/12 (85)	2/5 (40%)	0.29^a^
LA (cm^2^)	19.7 (16.5 to 21.7)	17.7 (15.2 to 19.8)	20.0 (18.6 to 24.8)	0.15^b^
LA-EDV/BSA (ml/m^2^)	32.0 (28.2 to 38.1)	30.0 (19.8 to 35.4)	31.6 (26.1 to 41.3)	0.32^b^
LA-EF (%)	65.9 (63.1 to 69.3)	67.1 (59.2 to 72.6)	59.0 (56.1 to 63.5)	0.09^b^
RA (cm^2^)	19.7 (17.9 to 22.2)	18.0 (15.2 to 21.1)	22.8 (17.1 to 26.4)	0.31^b^
RA-EF (%)	47.1 (39.6 to 59.9)	46.4 (36.3 to 59.2)	43.0 (25.7 to 60.2)	0.59^c^
LV-EDV (ml)	134.7 (122.1 to 173.5)	122.6 (97.7 to 141.9)	144.9 (135.3 to 198.7)	0.06^b^
LV-ESV (ml)	48.6 (42.5 to 60.6)	50.3 (37.0 to 60.8)	54.1 (49.2 to 79.2)	0.43^b^
LV-SV (ml)	87.0 (78.8 to 99.7)	66.2 (54.7 to 85.8)	93.4 (83.9 to 120.3)	**0.01 (1 vs. 2 *p *= 0.01; 2 vs. 3 *p *= 0.02)** ^b^
LV-EF (%)	64.4 (60.3 to 65.9)	61.1 (54.2 to 63.8)	60.1 (59.2 to 65.5)	0.11^b^
LV-M (g)	75.5 (59.9 to 104.9)	74.4 (65.0 to 81.7)	77.3 (74.9 to 120.9)	0.38^b^
RV-EF (%)	54.3 (52.0 to 57.9)	51.9 (46.2 to 59.2)	50.6 (46.6 to 56.7)	0.25^c^
RV-EDV (ml)	150.4 (133.5 to 181.1)	123.2 (111.1 to 151.7)	157.5 (149.4 to 218.5)	0.07^c^
RV-SV (ml)	82.3 (75.8 to 98.5)	69.9 (53.3 to 79.7)	82.9 (76.3 to 110.6)	**0.02 (1 vs. 2 *p *= 0.03)** ^c^
Global native T1 (ms)	1,024.1 (990.5 to 1,046.1)	1,013.7 (1,000.9 to 1,041.4)	1,004.1 (986.9 to 1,025.9)	0.50^b^
Global T2 (ms)	50.2 (49.0 to 52.3)	48.6 (47.0 to 50.4)	49.8 (49.2 to 51.7)	0.07^b^
Global ECV (%)	24.0 (23.4 to 26.7)	23.2 (22.3 to 26.5)	22.9 (21.4 to 25.1)	0.53^c^
Global longitudinal strain (%)	−18.8 (−19.9 to −17.7)	−16.8 (−18.6 to −15.2)	−19.3 (−21.1 to −16.5)	**0.04 (1 vs. 2 *p *= 0.04)** ^b^
Global radial strain (%)	26.0 (23.8 to 29.4)	23.4 (22.4 to 27.3)	25.5 (24.4 to 27.1)	0.28^b^
Global circumferential strain (%)	−16.7 (−18.1 to −15.7)	−15.7 (−17.3 to −15.2)	−16.6 (−17.3 to −16.0)	0.36^b^

LV-SV, left ventricular stroke volume; FU, follow-up; LA, left atrium; LA-EDV, left atrial end-diastolic volume; BSA, body surface area; LA-EF, left atrial ejection fraction; RA, right atrium; RA-EF, right atrial ejection fraction; LV-EDV, left ventricular end-diastolic volume; LV-ESV, left ventricular end-systolic volume; LV-EF, left ventricular ejection fraction; LV-M, left ventricular mass; RV-EF, right ventricular ejection fraction; RV-EDV, right ventricular end-diastolic volume; RV-SV, right ventricular stroke volume.

Values presented as median and IQR. A *p*-value <0.05 was considered significant; *p*-values in bold represent significant findings.

^a^ χ^2^ or Fisher's exact test.

^b^ Kruskal–Wallis test.

^c^ ANOVA.

### 3.5 Subgroups with and without late gadolinium enhancement on baseline scans

**Figure 3:**
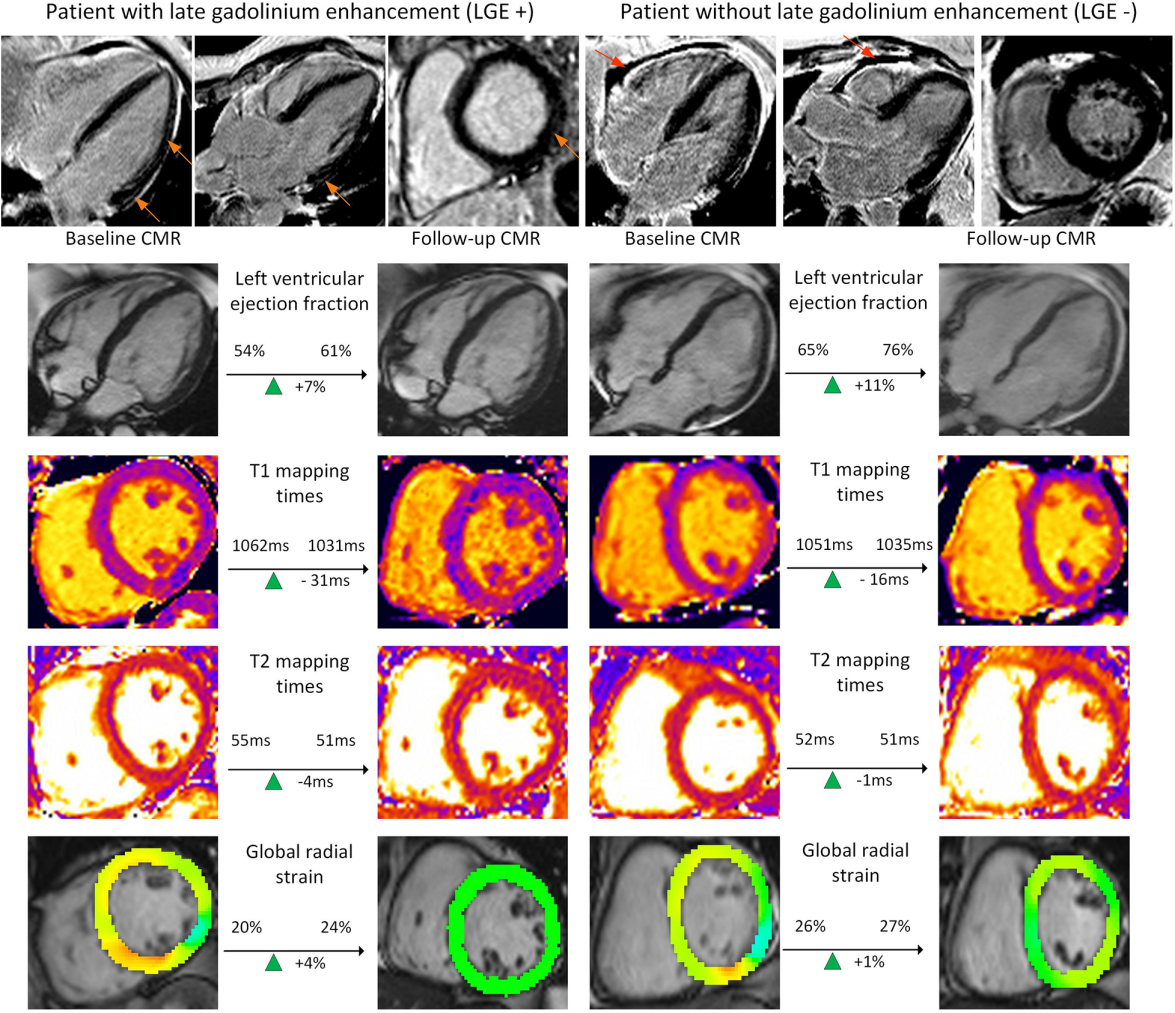
Examples of functional and tissue parameter changes in patients with and without LGE. Provided are examples for a patient with late gadolinium enhancement (LGE) (left columns) and one without (right columns). Images for ventricular function based on cine images, T1 and T2 mapping as well as post-processed cine images for global radial strain are presented (from top to bottom). These images are exemplary and do not represent changes in left ventricular stroke volume, the main finding of the study. Orange images point at subepicardial LGE. Red arrows point at a pericardial effusion.

For the 20 patients with and the 23 without LGE findings on baseline, FU analysis revealed improvement in global circumferential and radial strains (all *p* < 0.002). In the LGE negative (−) group, this was accompanied by an improvement in LV ejection fraction [baseline 64.0% (58.2%–65.5%) vs. FU 64.8% (63.1%–68.1%); *p* = 0.04] as well as a slight lower myocardial mass [baseline 72.5 g (59.9–78.8 g) vs. FU 70.8 g (63.8–84.4 g); *p* = 0.02]. Both baseline and FU values were within normal ranges. Myocardial tissue analysis by T1 and T2 mapping did not disclose any changes between baseline and FU scans for LGE positive (+) and (−) cohorts (Table 4). Figure 3 shows individual examples of patients with and without LGE and their individual trajectories.

**Table 4:** CMR results—subgroups with and without LGE on baseline scans.

Parameter	LGE (+) baseline (*N* = 20)	LGE (+) follow-up (*N* = 20)	*p*-value	LGE (−) baseline (*N* = 23)	LGE (−) follow-up (*N* = 23)	*p*-value
LA (cm^2^)	19.7 (16.7 to 21.4)	21.1 (18.0 to 24.7)	0.14^a^	18.7 (16.2 to 20.5)	19.4 (16.9 to 22.6)	0.59^a^
LA-EDV/BSA (ml/m^2^)	31.4 (24.3 to 36.8)	35.1 (27.6 to 39.3)	0.22^a^	31.3 (28.8 to 38.7)	32.3 (27.1 to 39.1)	0.78^a^
LA-EF (%)	65.7 (62.0 to 69.6)	65.8 (59.5 to 71.3)	0.65^a^	65.1 (60.3 to 69.6)	64.5 (60.0 to 67.8)	0.81^a^
RA (cm^2^)	20.1 (17.7 to 22.6)	20.9 (17.7 to 22.5)	0.91^aa^	18.4 (17.0 to 22.1)	18.8 (17.3 to 23.0)	0.37^a^
RA-EF (%)	46.8 (36.6 to 57.9)	45.5 (35.7 to 52.9)	0.89^b^	46.9 (39.5 to 60.6)	49.5 (39.9 to 55.9)	0.36^b^
LV-EDV (ml)	141.9 (126.8 to 175.0)	153.7 (125.4 to 167.4)	0.21^a^	128.7 (113.5 to 144.1)	129.2 (120.7 to 140.6)	0.31^a^
LV-ESV (ml)	53.9 (47.4 to 73.7)	58.8 (45.0 to 74.1)	0.97^a^	46.1 (41.4 to 59.5)	45.8 (41.2 to 52.2)	0.09^a^
LV-SV (ml)	90.6 (72.9 to 106.5)	91.7 (73.6 to 104.9)	0.33^a^	80.1 (69.4 to 92.3)	79.8 (77.2 to 89.2)	0.09^a^
LV-EF (%)	63.0 (58.8 to 65.1)	61.1 (59.0 to 68.7)	0.57^a^	64.0 (58.2 to 65.5)	64.8 (63.1 to 68.1)	**0.04** ^a^
LV-M (g)	78.7 (70.2 to 114.9)	83.8 (68.9 to 106.8)	0.72^a^	72.5 (59.9 to 78.8)	70.8 (63.8 to 84.4)	**0.02** ^a^
RV-EF (%)	53.6 (50.9 to 58.0)	55.3 (51.5 to 58.7)	0.53^b^	53.7 (49.3 to 57.6)	54.2 (51.5 to 56.6)	0.74^b^
RV-EDV (ml)	153.0 (132.9 to 182.1)	163.3 (138.6 to 186.0)	0.32^b^	145.1 (123.3 to 158.1)	139.4 (127.5 to 151.4)	0.95^b^
RV-SV (ml)	80.9 (73.8 to 102.5)	89.1 (65.9 to 103.6)	0.33^b^	78.6 (67.0 to 85.3)	72.5 (69.3 to 91.8)	0.81^b^
Global native T1 (ms)	1,013.2 (988.2 to 1,037.2)	1,007.3 (991.9 to 1,030.9)	0.55^a^	1,016.7 (1,003.0 to 1,040.9)	1,021.2 (989.4 to 1,035.3)	0.28^a^
Global T2 (ms)	49.9 (49.0 to 52.1)	49.6 (48.8 to 51.1)	0.28^a^	49.2 (48.4 to 51.6)	49.9 (48.5 to 51.4)	0.49^a^
Global longitudinal strain (%)	−18.7 (−19.3 to −17.2)	−18.3 (−19.7 to −16.6)	0.53^a^	−18.3 (−20.0 to −16.7)	−17.8 (−19.2 to −16.7)	0.22^a^
Global radial strain (%)	23.8 (22.2 to 26.8)	27.3 (23.5 to 28.8)	**0.003** ^a^	26.0 (24.8 to 29.9)	28.7 (26.0 to 34.6)	**0.004** ^a^
Global circumferential strain (%)	−15.7 (−17.1 to 15.0)	−17.0 (−17.7 to −15.7)	**0.01** ^a^	−16.7 (−18.3 to −16.3)	−17.8 (−19.9 to −16.8)	**0.01** ^a^

LGE, late gadolinium enhancement; LA, left atrium; LA-EDV, left atrial end-diastolic volume; BSA, body surface area; LA-EF, left atrial ejection fraction; RA, right atrium; RA-EF, right atrial ejection fraction; LV-EDV, left ventricular end-diastolic volume; LV-ESV, left ventricular end-systolic volume; LV-SV, left ventricular stroke volume; LV-EF, left ventricular ejection fraction; LV-M, left ventricular mass; RV-EF, right ventricular ejection fraction; RV-EDV, right ventricular end-diastolic volume; RV-SV, right ventricular stroke volume.

Values presented as median and IQR. A *p*-value <0.05 was considered significant; *p*-values in bold represent significant findings.

^a^ Wilcoxon test.

^b^ Paired Student’s *t*-test.

### 3.6 Subgroup comparison between asymptomatic and symptomatic patients on FU

In total, 22 patients were asymptomatic on FU compared with the 21 symptomatic patients on FU. The asymptomatic patients had lower left atrial function indexes (left atrial size in cm^2^ *p* = 0.049 and left atrial end-diastolic volume index divided by body surface area *p* = 0.01) and larger RV end-diastolic volume (*p* = 0.049) at baseline (Supplementary Table E1). On FU, only left atrial ejection fraction was shown to reveal lower values in asymptomatic patients (*p* = 0.01). Tissue characterization showed lower T2 values on FU between the two cohorts [T2 asymptomatic on FU 50.5 ms (49.2–51.6 ms) vs. symptomatic at FU 49.2 ms (48.4–50.8 ms); *p* = 0.04] (Supplementary Table E2). Comparing baseline and FU results for symptomatic patients at FU, LV-SV (*p* = 0.03) as well as global radial (*p* = 0.01) and circumferential strain (*p* = 0.02) showed improvement (Supplementary Table E2). Correspondingly, radial (*p* = 0.001) and circumferential strains (*p* = 0.002) improved in asymptomatic patients at FU as well (Supplementary Table E3).

## 4 Discussion

The principal results of this mid-term FU of symptomatic PCS patients in an outpatient setting are as follows: First, the majority of functional and tissue parameters remained stable even after a year. Second, the symptoms declined during mid-term FU. Third, LV-SV as well as myocardial deformation indexes, especially radial and circumferential strains, might potentially reflect a reverse remodeling. And lastly, this change is accompanied by a decrease in arterial blood pressure.

Cardiac post-acute sequalae of COVID-19 are manifold and include subacute myocarditis and chronic inflammation, ischemic tissue injury as well as new-onset arrhythmias and strokes (4, 10, 15). The exact pathophysiology is not entirely understood; however, a possible combination of mechanisms could play a role, such as direct viral toxicity, autoimmune responses, and inflammatory cascades. Hospitalized patients with COVID-19 have a high prevalence of non-ischemic and ischemic injuries, especially patients with elevated troponins (15). Therefore, elevated troponins might be associated with adverse cardiovascular outcomes (22). In contrast to this, outpatient cases without abnormalities on basic testing are less researched and followed-up. Hanneman et al. recently presented echocardiographic- and CMR-based data in symptomatic patients at 3–6 and 12–18 months FU after mild COVID-19 infection (23). The authors did not find structural or functional differences between the COVID-19 cohort and healthy controls; however, elevated cardiac T1 times were associated with symptoms at FU (23). A prospective trial by Puntmann et al. including 346 patients with COVID-19 infection reported baseline and FU scan results at a median of 109 days (IQR, 77–177 days) and 329 days (IQR, 274–383 days), respectively (10). Myocardial tissue characterization revealed overall lower T1 and T2 times as well as lower RV ejection fractions at FU. This change was concomitant with lower systolic blood pressure. Remarkably, patients with ongoing symptoms at FU had higher T2 times than asymptomatic patients. In contrast to this study, we report lower T2 times in symptomatic patients; however, all median mapping values in the current study are within normal ranges. As the observed differences are falling well into an intraobserver range, it is unlikely that the lower T2 values reflect a pathologic process (24). In contrast to the discrepancies regarding the T2 findings in the aforementioned study, our findings are in line regarding the lower systolic blood pressure. Unfortunately, SV was not reported by Puntmann et al. (10).

Following the importance of hospitalized vs. non-hospitalized patient groups, one should consider the timing of the CMR scan after the acute infection (4, 14). In a study by Zhang et al., 39 outpatients were scanned 26 days after an acute infection (25). In addition, CMR scans before the infection were available for an intraindividual comparison. The authors report no change in function, volume, and tissue properties. Although this study reports the acute phase, it underlines the mild effect of COVID-19 on the myocardium. Kravchenko et al. report a comparison of PCS patients, scanned at a median of 103 days after the infection, and a healthy cohort, with no significant differences regarding function and tissue characterization (13). Although no FU was carried out in this study, the overall results regarding tissue characteristics are in line with our findings.

Given the limited data on CMR studies, one should also look into other imaging modalities. A current echocardiography study by Olszanecka et al. reported FU data for 229 patients with mild to severe COVID-19 infection at 3, 6, and 12 months (26). They found a significant improvement in LV-SV as well as a reduction in LV mass accompanied by a lower systolic blood pressure at FU. These findings are in line with ours, promoting the importance of hemodynamic variables such as blood pressure. In contrast to these results, Young et al. carried out pre- and post-COVID-19 infection transthoracic echocardiography studies in 259 patients, reporting no statistically significant changes in the entire cohort except an improvement in LV ejection fraction after the infection (27). It should be noted that blood pressures did not change throughout the study, with systolic blood pressures being 130 and 132 mmHg at baseline and FU, respectively. This underlines the importance of hemodynamic factors.

Infections with COVID-19 might either worsen pre-existing arterial hypertension or even induce new-onset arterial hypertension based on interactions with the angiotensinogen II receptor (28, 29). It seems that blood pressure changes are related to autonomic dysfunction in these patients (30–32). The association between arterial blood pressure dysregulation has not only been proven for COVID-19 but also other viral infections (33, 34). The increased afterload might obstruct proper diastolic functioning, reducing LV-SV. An impaired ability to increase LV-SV during exercise has been established as a pathophysiologic link to dyspnea especially in heart failure with preserved ejection fraction (35, 36). The overlap extends to the observation that acute COVID-19 shows an association with this entity (37). Conversely, low LV-SV might even be able to better predict onset of heart failure than pure assessment of ejection fraction and therefore holds promising value especially in longitudinal FU (38, 39). Changes however might be small and within low-normal ranges, needing precise imaging tools, such as CMR, to be picked up (16). Whether this association transmits to PCS has yet to be established. It seems that risk factors, such as female gender, obesity, and arterial hypertension, predispose to PCS as well as heart failure with preserved ejection fraction (40, 41). Whether the underlying pathophysiologic mechanism, such as microvascular inflammation or autoimmune imbalances (42), autonomic dysfunction, commonly in the form of the postural orthostatic tachycardia syndrome in patients with PCS, is associated with impaired regulation of LV-SV, promoting the importance of this volumetric parameter (43).

The presented study results should be viewed under potential confounders that might as well impact the myocardium after an acute COVID-19 infection. These include potential alterations in lung function, especially in regard to pulmonary embolism, as well as after COVID-19 vaccine (14, 44). Although the effects of vaccines on the myocardium are often described as mild, some studies report tissue alterations in the form of scars or elevated T1 times (45). Lastly, the symptomatic overlap to a chronic fatigue syndrome (CFS) should be mentioned, which could be present in our patient cohort as well, given the common overlap between PCS and CFS (3, 46). These limitations warrant future prospective multi-center studies investigating the pathophysiologic links. Indices of mechanical ventricular function, for example circumferential, radial, and longitudinal strains, should be incorporated into such efforts as they might be valuable indicators for a recovery of the myocardium as shown across all subgroup comparisons in this study. In the LGE negative (−) subgroup, this was accompanied by a median reduction in LV mass, potentially influencing the deformation. Similar findings with a shorter FU were published recently based on CMR data (47). The authors reported lower strain rates at baseline; this finding is backed-up by recent evidence from echocardiography, suggesting that these changes already take place in patients shortly after an acute COVID-19 infection (48). Overall and in accordance with our findings, strain indexes seem to improve on FU (49). The same holds true for COVID-19 vaccination-associated changes (50). Certainly, the end-goal should be to couple volumetric and deformation indexes to treatment strategies in randomized controlled trials, such as the current ongoing MYOFLAME-19 study (NCT05619653) (51).

As the detected changes are rather small, direct implications for clinical practice are hard to infer from this analysis. However, a major point to be made is the association of dysregulated blood pressure and PCS (30). Evidence is accumulating that COVID-19 can trigger dysregulation of blood pressure responses, especially shortly after an acute infection (31). Focus on patient management should be on identification of co-existing diseases, such as arterial hypertension, diabetes mellitus, hyperlipidemia as well as heart failure with preserved ejection fraction. FU should be carried out in symptomatic patients, optimally with the same imaging technique and at the same site to precisely compare and track changes over time (16).

### 4.1 Limitations

The major limitation concerning this study is the retrospective nature of the analysis. As all patients were referred from outpatient providers, not all medical records were available, particularly laboratory and ancillary tests. The referral by outpatient providers might potentially introduce a referral bias as well as lack of data for patient characterization. Furthermore, not all patients underwent an FU scan with contrast media application, which was decided by the physician performing the exam. Lastly, the change in blood pressure over the time course of the study is a confounder for the LV-SV and strain results and our analysis has to be viewed under this circumstance.

## 5 Conclusion

Mid-term FU in patients with PCS reveals a reduction in symptomatic load accompanied by improved LV-SV as well as global LV deformation indexes. Blood pressure dysregulation, especially after the acute infection, is an important hemodynamic factor, which needs to be taken into consideration while assessing patients with PCS. Further multi-center research is needed on medical patient management as well as the interaction between autonomic dysfunction and PCS.

## Data Availability

The datasets analyzed during the current study are not publicly available due to German laws but are available from the corresponding author on reasonable request.
