# Supplementary material for: Trajectories of functional and structural myocardial parameters in post-COVID-19 syndrome—insights from mid-term follow-up by cardiovascular magnetic resonance
Source: Front Cardiovasc Med. 2024 Apr 2;11:1357349. doi: 10.3389/fcvm.2024.1357349 (PMC11018885; doi:10.3389/fcvm.2024.1357349)
Supplement: Supplementary file 1 [file Table1.docx]

Supplementary Material

Trajectories of functional and structural myocardial parameters in Post-COVID-19 Syndrome – insights from long-term follow-up by cardiovascular magnetic resonance

Jan Gröschel^1,2,3,4^, Leonhard Grassow^1,2,3^, Phillip van Dijck^1,4^, Yashraj Bhoyroo^1^, Edyta Blaszczyk^1,2,3^, Jeanette Schulz-Menger^1,2,3,*^

*** Correspondence:** Jeanette Schulz-Menger, jeanette.schulz-menger@charite.de

# Supplementary data E1. CMR image acquisition parameters

**Balanced steady-state free precession cine imaging:**

Long axis: ECG triggered with retrogating, repetition time (TR) 2.78 ms, 30 reconstructed phases, echo time (TE) 1.19ms, field of view (FOV) 340x276mm2, matrix 192x156, voxel size 1.8x1.8mm2, slice thickness (ST) 6mm, flip angle (FA) 74°, GRAPPA acceleration factor 2.

Short axis: ECG triggered with retrogating, TR 3.31ms, 30 reconstructed phases, TE 1.44ms, FOV 380x308.75mm2, matrix 192x156, voxel size 2.0x2.0mm2, ST 7mm, gap between slice: 0mm, FA 80°, GRAPPA acceleration factor 2.

**T2 mapping:**

Balanced steady-state free precession sequence, T2prep times 0, 25 and 55 ms, 3 recovery heartbeats, TR 2.98ms, TE 1.12ms, FA 70°, FOV 380×288mm2, matrix 224x170, voxel size 1.7x1.7mm2, ST 8mm, gap between slices 0mm, GRAPPA acceleration factor 2, motion corrected.

**T1 Mapping:**

5(3)3 MOLLI acquisition scheme, TR 3.9ms, TE 1.13ms, FA 35°, TI 180ms, FOV 360×270mm2, matrix 256x144, voxel size 1.4x1.4mm2, ST 8 mm, gap between slices 0mm, GRAPPA acceleration factor 2, motion corrected.

**Late gadolinium enhancement:**

PSIR reconstruction, gradient echo sequences, TI 240ms, TR 29.76ms, TE 5.17ms, FA 30°, FOV 350x263mm2, matrix 256x166, voxel size 1.4x1.4mm2, ST 7mm, gap between slices 0mm.

**Synthetic Extracellular volume:**

Prototype-sequence,

Pre-contrast: 5(3)3 MOLLI acquisition scheme, TR 3.9ms, TE 1.13ms, FA 35°, TI 180ms, FOV 360×270mm2, matrix 256x144, voxel size 1.4x1.4mm2, ST 6 mm, GRAPPA acceleration factor 2, motion corrected.

Post-contrast: 4(1)3(1)2 MOLLI acquisition scheme, TR 5ms, TE 1.13ms, FA 35°, TI 260ms, FOV 360×270mm2, matrix 256x144, voxel size 1.4x1.4mm2, ST 6 mm, GRAPPA acceleration factor 2, motion corrected.

# Supplementary data E2. Baseline and follow-up comparisons of symptomatic and asymptomatic patients

| Parameter | Asymptomatic at FU (N=22) | Symptomatic at FU (N=21) | p-value |
| --- | --- | --- | --- |
| LA (cm^2^) at baseline | 19.9 (16.7-26.6) | 18.0 (15.9-20.2) | **.049*** |
| LA -EDV/BSA (ml/m^2^) at baseline | 33.7 (29.1-41.4) | 29.6 (23.6-32.1) | **.011*** |
| LA-EF (%) at baseline | 63.1 (56.2-70,0) | 67.2 (64.7-69.3) | .06* |
| RA (cm^2^) at baseline | 20.5 (16.6-23.8) | 19.0 (17.8-21.4) | .50* |
| RA-EF (%) at baseline | 45.8 (34.9-58.5) | 47.6 (42.5-61.3) | .28^†^ |
| LV-EDV (ml) at baseline | 140.1 (123.8-174.0) | 131.2 (111.6-143.8) | .09* |
| LV-ESV (ml) at baseline | 52.0 (45.5-72.8) | 49.0 (39.2-56.1) | .12* |
| LV-SV (ml) at baseline | 90.7 (76.6-110.5) | 79.6 (69.6-90.4) | .09* |
| LV-EF (%) at baseline | 63.5 (57.7-65.8) | 62.6 (59.5-65.1) | .87* |
| LV-M (g) at baseline | 77.0 (69.5-123.3) | 75.1 (58.6-81.2) | .12* |
| RV-EF (%)at baseline | 53.6 (49.6-57.3) | 54.0 (51.6-58.2) | .40^†^ |
| RV-EDV (ml) at baseline | 154.6 (125.7-203.3) | 144.1 (122.0-159.9) | **.049^†^** |
| RV-SV (ml) at baseline | 84.7 (74.4-104.7) | 77.0 (71.8-81.5) | .07^†^ |
| Global native T1 (ms) at baseline | 1009.5 (980.8-1038.7) | (1016.9 (1001.2-1043.0) | .27* |
| Global T2 (ms) at baseline | 50.5 (48.8-52.3) | 49.3 (48.8-51.2) | .40* |
| Global ECV (%) at baseline | 23.6 (22.2-26.5) | 24.5 (23.1-26.7) | .63^†^ |
| Global longitudinal strain (%) at baseline | -183 (-19.0- (-16.7) | -19.1(-20.2-(-17.3) | .11* |
| Global radial strain (%) at baseline | 25.6 (22.6-28.6) | 24.7 (23.4-28.1) | .73* |
| Global circumferential strain (%) at baseline | -16.7 (-17.8 -(-15,3) | -16.2(-17.6-(-15.7) | .69* |
| LA (cm^2^) at FU | 21.5 (18.0-25.3) | 18.1 (16.8-21.9) | .05* |
| LA -EDV/BSA (ml/m^2^) at FU | 35.4 (31.5-40.9) | 32.1 (25.4-37.9) | .09* |
| LA-EF (%) at FU | 63.2 (58.9-65.5) | 67.7 (60.7-72.6) | **.01*** |
| RA (cm^2^) at FU | 20.4 (17.7-23.4) | 19.4 (16.4-22.4) | .36* |
| RA-EF (%) at FU | 44.0 (33.2-55.1) | 48.9 (40.6-55.6) | .26^†^ |
| LV-EDV (ml) at FU | 135.0 (126.2-183.1) | 137.3 (113.3-153.2) | .14* |
| LV-ESV (ml) at FU | 51.9 (42.8-74.2) | 47.8 (34.7-60.4) | .23* |
| LV-SV (ml) at FU | 84.4 (78.3-111.6) | 84.0 (71.7-97.7) | .22* |
| LV-EF (%) at FU | 64.1 (59.2-66.3) | 64.7 (60.3-69.6) | .31* |
| LV-M (g) at FU | 75.1 (68.3-108.8) | 74.7 (58.5-88.5) | .42* |
| RV-EF (%) at FU | 53.7 (50.7-55.2) | 55.8 (54.0-59.2) | .16^†^ |
| RV-EDV (ml) at FU | 143.5 (132.4-198.0) | 148.8 (126.4-172.4) | .45^†^ |
| RV-SV (ml) at FU | 79.3 (70.5-99.2) | 76.2 (65.9-97.4) | .40^†^ |
| Global native T1 (ms) at FU | 1008.1 (987.3-1028.3) | 1021.3 (1003.6-1033.9) | .16* |
| Global T2 (ms) at FU | 50.5 (49.2-51.6) | 49.2 (48.4-50.8) | **.04*** |
| Global ECV (%) at FU | 23.1 (21.1-24.8) | 23.1 (22.5-25.8) | .47^†^ |
| Global longitudinal strain (%) at FU | -17.8 (-18.9-(-16.5) | -18.5 (-19.6- (-16.9) | .22* |
| Global radial strain (%) at FU | 28.2 (25.4-34.0) | 27.1 (23.6-29.4) | .42* |
| Global circumferential strain (%) at FU | -17.6(-19.8 -(-16.6) | -17.0(-18.1-(-15.7) | .33* |

Note: FU= follow-up; LA=left atrium; LA-EDV=left atrial end diastolic volume; BSA=body surface area; LA-EF=left atrial ejection fraction; RA=right atrium; RA-EF=right atrial ejection fraction; LV-EDV=left ventricular end-diastolic volume; LV-ESV=left ventricular end-systolic volume; LV-SV=left ventricular stroke volume; LVEF=left ventricular ejection fraction; LV-M=left ventricular mass; RVEF=right ventricular ejection fraction; RV-EDV=right ventricular end-diastolic volume; RV-SV=right ventricular stroke volume. Values presented as median and IQR. *Kruskal-Wallis test; †ANOVA; a p-value <.05 was considered significant, p-values in bold represent significant findings.

# Supplementary material E3. Baseline and follow-up comparison of symptomatic patients

| Parameter | Symptomatic at FU - baseline (N=21) | Symptomatic at FU – FU (N=21) | p-value |
| --- | --- | --- | --- |
| LA (cm^2^) | 18.0 (15.9-20.2) | 18.1 (16.8-21.9) | .15* |
| LA -EDV/BSA (ml/m^2^) | 29.6 (23.6-32.1) | 32.1 (25.4-37.9) | .16* |
| LAEF (%) | 67.2 (64.7-69.3) | 67.7 (60.7-72.6) | .93* |
| RA (cm^2^) | 19.0 (17.8-21.4) | 19.4 (16.4-22.4) | .82* |
| RA-EF (%) | 47.6 (42.5-61.3) | 48.9 (40.6-55.6) | .63^†^ |
| LV-EDV (ml) | 131.2 (111.6-143.8) | 137.3 (113.3-153.2) | .16* |
| LV-ESV (ml) | 49.0 (39.2-56.1) | 47.8 (34.7-60.4) | .49* |
| LV-SV (ml) | 79.6 (69.6-90.4) | 84.0 (71.7-97.7) | **.03*** |
| LV-EF (%) | 62.6 (59.5-65.1) | 64.7 (60.3-69.6) | .08* |
| LV-M (g) | 75.1 (58.6-81.2) | 74.7 (58.5-88.5) | .10* |
| RV-EF (%) | 54.0 (51.6-58.2) | 55.8 (54.0-59.2) | .46^†^ |
| RV-EDV (ml) | 144.1 (122.0-159.9) | 148.8 (126.4-172.4) | .11^†^ |
| RV-SV (ml) | 77.0 (71.8-81.5) | 76.2 (65.9-97.4) | .14^†^ |
| Global native T1 (ms) | (1016.9 (1001.2-1043.0) | 1021.3 (1003.6-1033.9) | .36* |
| Global T2 (ms) | 49.3 (48.8-51.2) | 49.2 (48.4-50.8) | .66* |
| Global ECV (%) | 24.5 (23.1-26.7) | 23.1 (22.5-25.8) | .50^†^ |
| Global longitudinal strain (%) | -19.1(-20.2-(-17.3) | -18.5 (-19.6- (-16.9) | .30* |
| Global radial strain (%) | 24.7 (23.4-28.1) | 27.1 (23.6-29.4) | **.01*** |
| Global circumferential strain (%) | -16.2(-17.6-(-15.7) | -17.0(-18.1-(-15.7) | **.02*** |

Note:FU= follow-up; LA=left atrium; LA-EDV=left atrial end diastolic volume; BSA=body surface area; LA-EF=left atrial ejection fraction; RA=right atrium; RA-EF=right atrial ejection fraction; LV-EDV=left ventricular end-diastolic volume; LV-ESV=left ventricular end-systolic volume; LV-SV=left ventricular stroke volume; LVEF=left ventricular ejection fraction; LV-M=left ventricular mass; RVEF=right ventricular ejection fraction; RV-EDV=right ventricular end-diastolic volume; RV-SV=right ventricular stroke volume. Values presented as median and IQR. *Mann-Whitney-U; ^†^students t-test; a *p*-value <.05 was considered significant, *p*-values in bold represent significant findings.

# E4. Baseline and follow-up comparison of asymptomatic patients

| Parameter | Asymptomatic at FU - baseline (N=22) | Asymptomatic at FU – FU (N=22) | p-value |
| --- | --- | --- | --- |
| LA (cm^2^) | 19.9 (16.7-26.6) | 21.5 (18.0-25.3) | .59* |
| LA -EDV/BSA (ml/m^2^) | 33.7 (29.1-41.4) | 35.4 (31.5-40.9) | .88* |
| LAEF (%) | 63.1 (56.2-70,0) | 63.2 (58.9-65.5) | .96* |
| RA (cm^2^) | 20.5 (16.6-23.8) | 20.4 (17.7-23.4) | .29* |
| RA-EF (%) | 45.8 (34.9-58.5) | 44.0 (33.2-55.1) | .71^†^ |
| LV-EDV (ml) | 140.1 (123.8-174.0) | 135.0 (126.2-183.1) | .32* |
| LV-ESV (ml) | 52.0 (45.5-72.8) | 51.9 (42.8-74.2) | .38* |
| LV-SV (ml) | 90.7 (76.6-110.5) | 84.4 (78.3-111.6) | .38* |
| LV-EF (%) | 63.5 (57.7-65.8) | 64.1 (59.2-66.3) | .38* |
| LV-M (g) | 77.0 (69.5-123.3) | 75.1 (68.3-108.8) | .26* |
| RV-EF (%) | 53.6 (49.6-57.3) | 53.7 (50.7-55.2) | .77^†^ |
| RV-EDV (ml) | 154.6 (125.7-203.3) | 143.5 (132.4-198.0) | .84^†^ |
| RV-SV (ml) | 84.7 (74.4-104.7) | 79.3 (70.5-99.2) | .89^†^ |
| Global native T1 (ms) | 1009.5 (980.8-1038.7) | 1008.1 (987.3-1028.3) | .39* |
| Global T2 (ms) | 50.5 (48.8-52.3) | 50.5 (49.2-51.6) | .91* |
| Global ECV (%) | 23.6 (22.2-26.5) | 23.1 (21.1-24.8) | .71^†^ |
| Global longitudinal strain (%) | -183 (-19.0- (-16.7) | -17.8 (-18.9-(-16.5) | .40* |
| Global radial strain (%) | 25.6 (22.6-28.6) | 28.2 (25.4-34.0) | **.001*** |
| Global circumferential strain (%) | -16.7 (-17.8 -(-15,3) | -17.6(-19.8 -(-16.6) | **.002*** |

Nore: FU= follow-up; LA=left atrium; LA-EDV=left atrial end diastolic volume; BSA=body surface area; LA-EF=left atrial ejection fraction; RA=right atrium; RA-EF=right atrial ejection fraction; LV-EDV=left ventricular end-diastolic volume; LV-ESV=left ventricular end-systolic volume; LV-SV=left ventricular stroke volume; LVEF=left ventricular ejection fraction; LV-M=left ventricular mass; RVEF=right ventricular ejection fraction; RV-EDV=right ventricular end-diastolic volume; RV-SV=right ventricular stroke volume. Values presented as median and IQR. *Wilcoxon test; ^†^paired students t-test; a *p*-value <.05 was considered significant, *p*-values in bold represent significant findings.
